# Subgingival Prevalence and Antibiotic Susceptibility of Selenomonas noxia

**DOI:** 10.7759/cureus.83171

**Published:** 2025-04-29

**Authors:** Thomas E Rams, Charles E Hawley, Eugene J Whitaker

**Affiliations:** 1 Department of Periodontology and Oral Implantology, Temple University School of Dentistry, Philadelphia, USA; 2 Department of Periodontology, Tufts University School of Dental Medicine, Boston, USA; 3 Department of Restorative Dentistry, Temple University School of Dentistry, Philadelphia, USA

**Keywords:** antibiotics, dna probes, periodontal pocket, periodontitis, selenomonas noxia

## Abstract

Background

*Selenomonas noxia* is a putative periodontal pathogen in subgingival biofilm communities. This study compared the subgingival prevalence and levels of *S. noxia *in those with severe periodontitis and those with periodontal health and assessed the in vitro antibiotic susceptibility of the species.

Methods

Subgingival biofilm samples from 206 adults with stage III (severe) periodontitis and 48 adults with periodontal health were examined with an *S. noxia*-specific whole chromosomal nucleic acid probe. Minimum inhibitory concentration (MIC) values of six antibiotics against *S. noxia* were determined in vitro with antibiotic gradient strips.

Results

*Selenomonas noxia* was more frequently detected in patients with severe periodontitis (53.4%) than persons with periodontal health (20.8%) (2.6-fold more frequent, p = 0.0001, Fisher's exact test). Heavy subgingival colonization by *S. noxia* (≥10^6^ cells/subject subgingival specimen) was also more frequent in persons with severe periodontitis (16.0%) than those with periodontal health (2.1%) (7.6-fold more frequent, p = 0.008, Fisher's exact test). *Selenomonas noxia* was susceptible in vitro to amoxicillin, azithromycin, clindamycin, doxycycline, and metronidazole (all MIC values ≤ 0.75 mg/L) but resistant to spiramycin (MIC > 32 mg/L).

Conclusions

*Selenomonas noxia* was significantly more prevalent at significantly higher subgingival levels in patients with severe periodontitis than adults with periodontal health. However, heavy *S. noxia* subgingival colonization was present in only a subset of severe periodontitis patients and rarely in those with periodontal health. *Selenomonas noxia* was susceptible in vitro to several antibiotics of potential therapeutic use in periodontitis therapy, with the exception of spiramycin. *Selenomonas noxia *may contribute to periodontitis in patients harboring high subgingival numbers of the organism.

## Introduction

A diverse community of key pathogenic bacteria and herpesviruses are present in dysbiotic subgingival biofilms and inflamed gingiva in severe human periodontitis [1]. *Selenomonas noxia*, which may participate as a periodontal pathogen in periodontal pockets [2], is a small (1.1 µm in diameter by 1.1 to 3.2 µm long), gram-negative, obligately anaerobic, non-spore-forming, curved, crescent-shaped, motile rod that tumbles with flagella arranged in tufts on the concave side of the cell [3]. *Selenomonas noxia* is primarily detected in supra- and subgingival tooth biofilms, saliva, and gingival crevicular fluid [3-5], but it also may be recovered from other oral surfaces [5] and the gastrointestinal tract in some individuals [6]. *Selenomonas noxia* is undetectable or in low numbers at healthy periodontal sites [4,7], but increases in prevalence and abundance with increases in gingival tissue inflammation [7]. Higher subgingival levels of *S. noxia* are found in adults with periodontitis [2,4,7], but the organism still generally comprises only low mean proportions of the total subgingival microbiota in both periodontally healthy (0.7%) and established periodontitis sites (<1.5%) [7].

Unlike most other subgingival bacteria, *S. noxia* is significantly increased at the initial onset of human periodontitis disease activity [8,9], with *S. noxia* averaging 5.9% in the cultivable subgingival microbiota of seven (77.8%) of nine disease-active interproximal tooth surfaces suffering periodontal breakdown from a state of periodontal health [9]. In untreated established periodontitis sites, higher cultivable levels of subgingival *S. noxia *showed a positive correlation coefficient of 0.72 with progressive periodontal attachment loss [10].

*Selenomonas noxia* may also contribute to periodontitis treatment failure, as a significantly higher post-treatment frequency of subgingival *S. noxia* is associated with progressive periodontal attachment loss in patients experiencing poor outcomes after completion of conventional mechanical/surgical periodontal treatment augmented with either systemic metronidazole/amoxicillin or tetracycline drug therapy [11,12]. In contrast, only a low subgingival frequency of *S. noxia*, similar to levels found in periodontal health, is found in patients with successful periodontal therapy outcomes [11].

*Selenomonas noxia* is considered an “outlier species” in subgingival biofilms since in microbial cluster and community ordination analysis, it did not statistically cluster with other evaluated bacteria in their subgingival colonization patterns and could not be assigned to any of the five microbial cluster complexes identified in periodontal sites [2]. *Selenomonas noxia* was alone among 40 oral bacterial species tested in saliva that was statistically linked to women suffering from excess body weight/obesity [13]. More recently, the prevalence of salivary *S. noxia* was found to be significantly greater in overweight and obese persons than individuals with normal body mass index values [14]. Additional study of oral* S. noxia *may thus be justified because of its apparent association with periodontal pockets independent of other predominant subgingival bacteria [2] and its significant relationship with periodontitis disease activity and overweight/obese individuals [8-14]. This study further assessed the prevalence of *S. noxia *in adults with severe periodontitis as compared to those with periodontal health and evaluated the in vitro antibiotic susceptibility of *S. noxia *to six antibiotics of potential use in periodontitis therapy.

## Materials and methods

A total of 206 adults (97 males, 109 females; aged 36-84 years), diagnosed with stage III (severe) periodontitis [15] by periodontists in the United States in private dental practices, were included in the present study as their subgingival biofilm samples were consecutively received and processed with their informed consent for microbiological analysis by the Oral Microbiology Testing Service (OMTS) Laboratory, Temple University School of Dentistry, Philadelphia, PA. The periodontists based their diagnosis of severe periodontitis on comprehensive clinical and radiographic examination findings consistent with the American Academy of Periodontology/European Federation of Periodontology diagnostic criteria [15]. Persons identified with aggressive (molar-incisor pattern) periodontitis, acute necrotizing ulcerative gingivitis, or antibiotic use within the past 6 months, were excluded. Another 48 consenting adults (22 males, 26 females; aged 23-41 years) with periodontal health, according to the American Academy of Periodontology/European Federation of Periodontology diagnostic criteria [15], were also sampled by a single periodontist [16]. These systemically healthy persons each had ≥24 teeth, probing depths ≤4 mm, no clinical periodontal attachment loss, and bleeding on probing either absent or localized to only ≤5% of periodontal sites. The Temple University Institutional Review Board classified this study (IRB protocol no. 13442) as exempt since it involved secondary analysis of preexisting de-identified subgingival biofilm specimens [16] with *S. noxia *DNA probe testing and did not have any study subject-investigator contact or interaction.

Subgingival biofilm specimens

*Selenomonas noxia* testing was performed on subgingival biofilm specimens obtained in a previous study [16] prior to treatment from three deep (>6 mm) periodontal pockets with bleeding on probing on separate teeth in severe periodontitis patients and from three interproximal molar sites without bleeding on probing on separate teeth in persons with periodontal health. These sites were selected to represent the more severely affected sites in severe periodontitis patients and to provide an adequate amount of subgingival biofilm for testing in persons with periodontal health. The subgingival samples were obtained with sterile absorbent paper points (Johnson & Johnson, East Windsor, NJ, USA), as previously described [16]. The paper points were pooled per study subject into a plastic vial containing 500 µL of TE buffer (10 mM Tris-Cl, pH 7.5, 1 mM EDTA) and transported within 24 hours to the OMTS Laboratory at Temple University, where the vials were vortexed to dislodge bacteria from the paper points and kept at -80°C until laboratory testing was performed. The OMTS Laboratory was licensed for high-complexity bacteriological analysis by the Pennsylvania Department of Health and certified by the United States Centers for Medicare and Medicaid Services as adherent with Clinical Laboratory Improvement Amendments (CLIA) standards required of clinical laboratories engaged in diagnostic testing of human specimens in the United States. The OMTS Laboratory personnel performing the *S. noxia* laboratory procedures were blinded to the clinical status of the study subjects and their inclusion in the present study.

*Selenomonas noxia* DNA probe construction and validation

*Selenomonas noxia* type strain 43541 was obtained from the American Type Culture Collection (ATCC) (Manassas, VA, USA) and grown to purity on enriched Brucella blood agar, which was comprised of 4.3% Brucella agar supplemented with 0.3% bacto-agar, 5% defibrinated sheep blood, 0.2% hemolyzed sheep red blood cells, 0.0005% hemin, and 0.00005% menadione, and incubated anaerobically at 37ºC for seven days.

A digoxigenin-labeled, whole-chromosomal, DNA probe to detect *S. noxia* ATCC 43541 was constructed using the Genius™ Non-radioactive DNA Labeling and Detection Kit (Boehringer Mannheim Biochemicals, Indianapolis, IN, USA), following manufacturer instructions and previous descriptions [16]. The constructed *S. noxia *DNA probe was tested against freshly-grown colonies of *S. noxia *ATCC 43541, as well as a panel of 18 other oral bacterial species from the ATCC and eight fresh clinical isolates of oral bacterial species from the OMTS Laboratory, as previously described [16]. Probe detection threshold limits (sensitivity) were determined by adjusting suspensions of *S. noxia* ATCC 43541 to a 0.5 McFarland turbidity standard (approximately 1.5 x 10^8^ colony-forming-units [CFU]/mL) and applying dilutions to membranes to enable probe testing of cell numbers ranging from 10^3^-10^8^ CFU/mL.

In DNA probe performance testing, the constructed *S. noxia *probe provided a strong and unambiguous signal color reaction at a ≥10^4^ *S. noxia* cell count level, without any consistent cross-reactions with other periodontal taxa. A faint cross-reaction was found on occasion to very high (>10^8^) cell counts of other *Selenomonas* species but not with other periodontal taxa tested. Based on this, the signal color intensity comparable to 10^4^ *S. noxia* cells in positive control testing was used to identify *S. noxia*-positive test reactions among study subject subgingival biofilm specimens.

*Selenomonas noxia* DNA probe testing of subgingival specimens

Subgingival biofilm specimens per each study subject, 10^4^ and 10^6^ cells/mL of *S. noxia* ATCC 43541 as a positive control, and *Prevotella buccae *ATCC 33574 and TE buffer alone as negative controls were dot-blot applied to the surfaces of positively charged Zeta-Probe nylon membranes (Bio-Rad Laboratories, Richmond, CA, USA). Bacterial DNA extraction, denaturation, and hybridization with the heat-denatured *S. noxia* DNA probe, as well as probe detection, were performed as previously described [16]. Colorimetric DNA probe detection reactions were visually read and semi-quantitatively scored by comparing the intensity of the color development of the subgingival study subject specimens with those of the *S. noxia* positive controls at 10^4^ and 10^6^ cell concentrations on the same membrane [16].

*Selenomonas noxia* in vitro antibiotic testing

In vitro antibiotic gradient strip testing was used to determine the minimum inhibitory concentration (MIC) of six antibiotics against *S. noxia*. Pure cell suspensions of *S. noxia *ATCC 43541 were prepared and adjusted to a 0.5 McFarland turbidity standard using sterile physiologic saline and streaked with sterile cotton-tipped swabs onto enriched Brucella blood agar plates. After drying, predefined antibiotic gradient strips for amoxicillin, azithromycin, clindamycin, and metronidazole (Etest®, bioMérieux, Durham, NC, USA), as well as for doxycycline and spiramycin (MTS™, Lidofilchem SRL, Abruzzi, Italy), were applied in duplicate onto the inoculated media surfaces. After 48 hours of anaerobic incubation at 37ºC, the intersection between the border of *S. noxia* growth and the antibiotic gradient strip drug scale was read to determine in vitro MIC values, following the manufacturer’s instructions. Since no *S. noxia*-specific MIC interpretative guidelines for the tested antibiotics were available, the antibiotic susceptibility of *S. noxia* was determined using clinical susceptibility and resistance breakpoint values for amoxicillin, clindamycin, and metronidazole against *Prevotella *species, another gram-negative anaerobic rod frequently found in the oral cavity, as issued by the European Committee on Antimicrobial Susceptibility Testing [17], and general MIC breakpoints for azithromycin, doxycycline, and spiramycin, as recommended by the French Society for Microbiology [18]. MIC values less than or equal to the antibiotic susceptibility breakpoint concentration were classified as susceptible, with those more than or equal to antibiotic resistance breakpoint concentrations identified as resistant. *Bacteroides thetaiotaomicron* ATCC 29741 was used as a quality control strain in the antibiotic gradient strip susceptibility testing.

Data analysis

The prevalence of detectable subgingival *S. noxia *per study subject and the frequency of study subjects with heavy subgingival colonization by *S. noxia* (≥10^6^ cells/subject subgingival specimen) were tabulated, and differences between patients with severe periodontitis and those with periodontal health were assessed using Fisher’s exact test and a p-value of ≤0.05 for statistical significance. The data analysis was performed using the STATA/SE 17.0 for Windows (StataCorp, College Station, TX, USA) 64-bit statistical software package.

## Results

**Figure 1:**
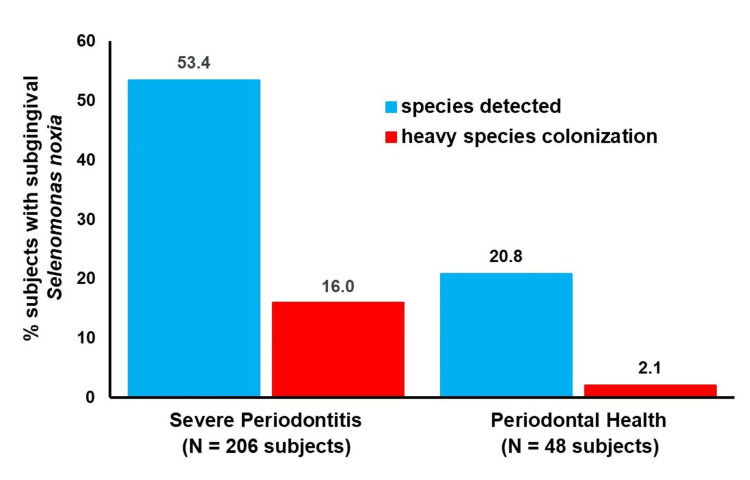
Distribution of Selenomonas noxia by subgingival abundance levels in severe periodontitis and periodontal health Species detected = *S. noxia* present at ≥10^4^ cells/subject subgingival specimen threshold; heavy species colonization = *S. noxia* present at ≥10^6^ cells/subject subgingival specimen threshold.

Table 1 and Figure 1 show the prevalence of detectable and heavy colonization by subgingival *S. noxia* in the study subject groups.

**Table 1:** Prevalence of detectable and heavy colonization by subgingival Selenomonas noxia in severe periodontitis and periodontal health Data presented as number and percentage of tested study subjects. - = *S. noxia *not detected; + = *S. noxia* detected; ++ = heavy *S. noxia *subgingival colonization ^a^Fisher's exact test, p-value ≤ 0.05 required for statistical significance between study subject groups. ^b^Number of *S. noxia* cells/pooled study subject subgingival biofilm specimen.

*S. noxia* cell counts	Subject groups, no. (%) subjects		Exact chi-square value	p-value^a^
	Severe periodontitis (N = 206 subjects)	Periodontal health (N = 48 subjects)		
-	96 (46.6)	38 (79.2)		
+ (10^4^-10^5^ cells)^b^	77 (37.4)	9 (18.7)	0.0171	0.0170
++ (≥10^6^ cells)^b^	33 (16.0)	1 (2.1)	0.0084	0.0080
Total + (≥10^4^ cells)^b^	110 (53.4)	10 (20.8)	0.0	0.0001

*Selenomonas noxia* DNA probe-positive reactions (≥10^4^ cells/subject sample) were detected in 110 (53.4%) of 206 severe periodontitis patients, a detection prevalence significantly greater than in persons with periodontal health, where *S. noxia* was detected in 10 (20.8%) of 48 individuals (2.6-fold higher proportions, p = 0.0001, Fisher's exact test). Heavy subgingival colonization by *S. noxia *(≥10^6^ cells/subject subgingival specimen) was also more frequent in severe periodontitis (33 patients; 16.0%) than persons with periodontal health (1 subject; 2.1%) (7.6-fold greater proportions, p = 0.008, Fisher's exact test).

Table 2 lists the in vitro susceptibility of *S. noxia* to six test antibiotics.

**Table 2:** Susceptibility in vitro of Selenomonas noxia to selected antibiotics MIC, minimal inhibitory concentration; S, susceptible to antibiotic; R, resistant to antibiotic

Antibiotic (susceptibility / resistance breakpoints, mg/L)	MIC against *S. noxia*, mg/L	*S. noxia* susceptibility
Amoxicillin (≤0.25 / >0.25)	0.047	S
Azithromycin (≤0.5 / >4)	<0.016	S
Clindamycin (≤0.25 / >0.25)	0.016	S
Doxycycline (≤4 / >8)	0.75	S
Metronidazole (≤4 / >16)	<0.016	S
Spiramycin (≤1 / >4)	>32	R

*Selenomonas noxia *was susceptible in vitro to amoxicillin, azithromycin, clindamycin, doxycycline, and metronidazole, with all antibiotic MIC concentration values ≤ 0.75 mg/L, but was resistant to spiramycin, with an MIC value of >32 mg/L.

## Discussion

Because *S. noxia *is difficult to recover by microbial culture [10,19], a molecular method was used in the present study to detect and quantitate *S. noxia* in subgingival biofilms. The whole chromosomal DNA probe constructed to detect *S. noxia* was comparable to probes in prior studies [10,19], where similarly constructed whole genomic probes to detect *S. noxia* were species-specific and did not cross-hybridize with other bacteria tested, including closely related *Selenomonas* and *Centipeda* species.

The present study found detectable subgingival *S. noxia* in severe periodontitis patients at a significantly greater prevalence than persons with periodontal health (2.6-fold higher proportions) (Table 1, Figure 1). Approximately one-half of severe periodontitis patients tested were DNA probe-positive for subgingival *S. noxia*. These findings are in agreement with and confirm results from several prior culture and molecular studies detecting *S. noxia* in periodontitis patients [2,4,7-12].

Moreover, a significantly greater frequency of heavy subgingival colonization by *S. noxia* (≥10^6^ cells/subject subgingival specimen) was found in severe periodontitis patients than persons with periodontal health (7.6-fold greater proportions) (Table 1, Figure 1). The prevalence of heavily colonized severe periodontitis study subjects (N = 33, 16.0%) is similar to the 23 (15.8%) of 146 periodontitis patients and the 75 (21.1%) of 356 periodontitis patients with heavy subgingival *S. noxia* colonization identified in China and Thailand, respectively [20,21]. This suggests that approximately one out of five to six severe periodontitis patients may potentially harbor high numbers of subgingival *S. noxia*.

In periodontally healthy adults, the subgingival detection of *S. noxia* in 10 (20.8%) of 48 adults with periodontal health confirms data from Tanner et al. [9], where 3 (21.4%) of 14 periodontally healthy subjects yielded cultivable *S. noxia *from subgingival sites. Heavy subgingival colonization by *S. noxia* was rare among persons with periodontal health in the present study, with only one (2.1%) of 48 periodontally healthy adults yielding high subgingival *S. noxia* counts (Table 1, Figure 1).

Consistent with our *S. noxia *DNA probe-based findings, serum IgG antibody titers to *S. noxia*, which likely reflect *S. noxia* oral colonization, are significantly higher in periodontitis patients than persons without periodontitis [22]. Similarly, high serum IgG antibody levels to *S. noxia* are more frequently found in periodontitis patients refractory to a combination of modified Widman flap surgery and systemic tetracycline therapy as compared to successfully treated patients and persons with periodontal health [23].

*Selenomonas noxia* was susceptible in vitro to several orally administered antibiotics of potential therapeutic use in periodontal therapy, including amoxicillin, azithromycin, clindamycin, doxycycline, and metronidazole, but not spiramycin (Table 2). These findings are in agreement with data from Moore et al. [3], where *S. noxia* was susceptible to clindamycin, erythromycin, penicillin, and tetracycline. The antibiotic susceptibility of *S. noxia* to azithromycin and metronidazole and its resistance in vitro to spiramycin, as determined in the present study, are previously unreported and expand knowledge on the antibiotic susceptibility profile of *S. noxia*. The molecular basis for in vitro drug resistance of *S. noxia* to spiramycin was not determined but may involve the acquisition of antibiotic resistance genes by *S. noxia* via mobile plasmids and/or conjugative transposons from other gram-negative microorganisms.

The antibiotic susceptibility of *S. noxia* is clinically relevant since conventional mechanical/surgical periodontal treatment may fail to adequately suppress *S. noxia* in periodontal pockets, increasing the risk of progressive/refractory periodontitis [11,12]. Both non-surgical periodontal therapy [24,25] and osseous periodontal surgery with apically repositioned flap placement [25] failed to significantly reduce subgingival *S. noxia* in severe periodontitis patients at three months post-treatment, which suggests a potential need for adjunctive antimicrobial chemotherapy for periodontitis patients with heavy subgingival colonization by *S. noxia*. The present *S. noxia* antibiotic susceptibility findings may aid in the application of recent clinical practice guidelines from the European Federation of Periodontology, which propose the use of adjunctive systemic and locally applied antimicrobials as possible interventions before periodontal surgery is considered in the treatment of periodontitis [26].

The potential role of *S. noxia* in the etiopathogenesis of periodontitis and non-oral medical disorders has received relatively little study. *Selenomonas noxia* ferments glucose into high levels of propionic acid and acetic acid [3] potentially toxic to gingival epithelial cells [27] and produces high levels of cytotoxic and malodorous hydrogen sulfide [28], which may collectively contribute to periodontal tissue breakdown. Goodson et al. hypothesized *S. noxia* oral colonization as a potential marker of pro-inflammatory host reactions related to the development of obesity, as a salivary *S. noxia* level of > 1.05% was alone able to successfully classify 98.4% of 313 females as being overweight/obese [13]. Williams et al. suggested that *S. noxia* potentially contributes to overweight/obesity by significantly increasing calorie extraction from normal dietary intake by facilitating digestion of cellulose and other normally non-digestible fibers in food [14]. In a multivariable analysis of United States National Health and Nutrition Examination Survey (NHANES) III data, where a multitude of potential sociodemographic, behavioral, and medical risk factors were controlled in the statistical analysis, individuals with high serum IgG antibody titers to *S. noxia* plus six other periodontal microorganisms ranking in the top tertile of antibody levels experienced an 86% higher risk of death due to diabetes-related causes over a 27-year period [29]. *Selenomonas noxia *of presumed oral origin and disseminating into the gastrointestinal tract of hepatitis C-affected patients with elevated serum albumin and bilirubin levels was one of only two bacterial species in the fecal microbiome to be significantly associated with a poor liver prognosis and decreased patient survival [6]. Additional research is needed to further delineate the potential relationship of *S. noxia* to the onset and progression of human periodontitis, inflammation-related systemic disorders such as obesity and diabetes, and hepatitis C-related liver disease. Greater attention may need to be given to the presence and levels of *S. noxia* in dysbiotic subgingival biofilms during periodontal treatment planning to enhance periodontal therapy outcomes in heavily infected species-positive patients and mitigate potential adverse extraoral effects of *S. noxia*.

The present study has several limitations that need to be considered. Greater sensitivity in subgingival *S. noxia* detection may have been obtained by sampling a greater number of periodontal sites per study subject and using a targeted molecular method, such as quantitative PCR, with a lower detection threshold level for *S. noxia* [5]. Untargeted approaches, such as high-throughput next-generation sequencing (NGS), may be less effective with low-abundance species such as *S. noxia*, depending on the reference library employed in bioinformatic data analysis [6]. No identification was made of other microbial species accompanying *S. noxia* in subgingival plaque biofilms. Clinical attachment loss measurements were not reported by the practicing periodontists who evaluated the severe periodontitis study subjects, but their identification of deep probing depths of >6 mm with bleeding on probing on three separate teeth in each of the patients, which strongly correlates (94.1% positive predictive value) with the presence of severe periodontal attachment loss in adults [30], supports their periodontal diagnosis of stage III (severe) periodontitis [15]. The cross-sectional study design precluded prospective clinical assessments of the patients. As a result, it is not known whether the 10 *S. noxia*-positive persons with periodontal health subsequently suffered clinical conversion to periodontitis, particularly the one periodontally healthy individual with heavy *S. noxia *subgingival colonization (Table 1, Figure 1), as suggested in prior studies where elevated levels of subgingival *S. noxia* were significantly associated with periodontally healthy sites longitudinally developing periodontal attachment loss [8,9].

## Conclusions

*Selenomonas noxia* was significantly more prevalent at significantly higher subgingival levels in patients with severe periodontitis than adults with periodontal health. However, heavy *S. noxia* subgingival colonization was present in only a subset of severe periodontitis patients and rarely in periodontal health. *Selenomonas noxia* was susceptible in vitro to several antibiotics of potential therapeutic use in periodontitis therapy, with the exception of spiramycin. *Selenomonas noxia* may contribute to periodontitis and some non-oral medical disorders in patients harboring high subgingival numbers of the organism.
